# Prevention and Management of Urolithiasis With Parsley and Barley Among the Hail Population, Saudi Arabia: Is It Fact or Not?

**DOI:** 10.7759/cureus.71990

**Published:** 2024-10-21

**Authors:** Hadi A Aldarwish, Akram Bokhari, Muath M Alshammari, Abdulrahman S Alshammari, Abdulelah G Alharbi, Sultan S Alsabhan, Yomna K Altwiher, Reem A Aleraidi, Anwar E Almallahi, Muath M Alshammari

**Affiliations:** 1 Urology, King Fahad Specialist Hospital, Dammam, SAU; 2 Urology, College of Medicine, University of Hail, Hail, SAU; 3 College of Medicine, University of Hail, Hail, SAU; 4 Surgery, College of Medicine, University of Hail, Hail, SAU

**Keywords:** barely, parsley, renal stone, saudi arabia, urolithiasis

## Abstract

Background: Urolithiasis is one of the most common urinary tract diseases. It is a multifactorial condition involving both environmental and metabolic aspects. Dietary changes and lifestyle modifications are crucial for managing and preventing urolithiasis. Barley and parsley have been utilized as a complementary treatment to manage urolithiasis, which may be helpful in managing and preventing this disease.

Methodology: This was a community-based cross-sectional study conducted to determine the effects of parsley and barley in managing urolithiasis among the population of Hail, Saudi Arabia by an electronic questionnaire. All responses were kept confidential. Statistical analyses were conducted using SPSS version 22 (Chicago, IL: SPSS Inc.).

Results: A total of 395 participants completed the questionnaire, with ages ranging from 18 years to more than 60 years; nearly half of them were females. Only 37 (9.4%) had a history of renal disease but 182 (46.1%) had a family history of renal stones. Regarding the frequency of barley and parsley used in the management of renal stones, 162 (41%) respondents used barley, while 176 (44.6%) of them used parsley to treat or prevent kidney stones. Factors associated with the use of barley and parsley in the management of renal stones were older age, female gender, non-healthcare staff, those with renal disease history, and family history of renal stones.

Conclusion: The results showed that among the Hail population, non-conventional therapies are more commonly used for treating renal stones than medical treatment. There were multiple factors associated with using barley and parsley to manage urolithiasis. Additional research is needed to gain a clearer understanding of the safety and effectiveness of non-conventional therapies, such as barley and parsley, in managing urolithiasis.

## Introduction

Urolithiasis, the formation of urinary stones, is one of the most common urinary tract diseases [1]. It is a multifactorial disease involving both environmental and metabolic aspects [2]. Multiple risk factors can contribute to the formation of urolithiasis, including age, gender, ethnicity, local climate, dietary habits, physical activity, and occupation [3]. A study by Safdar et al. showed that individuals in Saudi Arabia are particularly susceptible to developing urolithiasis, being two and a half times more likely to do so compared to others [4]. This increased risk is likely due to the region's hot climate.

Dietary changes and lifestyle modifications play a significant role in managing and preventing urolithiasis. Studies have shown that patients who adhere to dietary modifications and medical therapy can achieve remission rates of up to 90% [5,6]. The general recommendations for all renal stone formers include adequate hydration, lower protein intake, and increased vegetable consumption. Individuals with a history of kidney stones can significantly improve their chances of preventing recurrence and managing the condition effectively by implementing these dietary and lifestyle changes [7].

The Saudi population has a belief in utilizing complementary treatments to manage urolithiasis [8]. Among these, barley and parsley are widely available in the country, and may be helpful in managing urolithiasis [9]. Therefore, this study aimed to determine the utilization of barley and parsley for managing urolithiasis among the population of Hail.

## Materials and methods

Research design and setting

This was a community-based cross-sectional study conducted to investigate the effects of parsley and barley in managing urolithiasis (kidney stones) among the population of Hail, Saudi Arabia. The study protocol was approved by the Research Ethics Committee at the University of Hail (approval number H-2022-031). The research was carried out between March and August 2024. The researchers used an electronic questionnaire written in Arabic to collect data, which was distributed through various social media platforms (mainly WhatsApp and X) (table in appendix). All information gathered during the study was kept confidential in compliance with relevant privacy policies. The study was conducted in accordance with the ethical principles outlined in the Declaration of Helsinki, which provides guidelines for research involving human participants.

Sample size

The sample size was calculated using the World Health Organization's (WHO) prevalence rate calculation formula: ss = (Z^2^ × p × q)/d^2^, where ss = sample size, Z = 1.96 (the standard normal deviate at 95% confidence level), p = 0.5 (assumed prevalence rate of 50%), q = (1-p) = 0.5, and d = 0.05 (desired margin of error, 5%). According to this formula, the optimal sample size for this study was 384, which would achieve a ±5% accuracy with a 95% confidence interval (CI). However, the study ultimately included 395 participants. The inclusion criteria were participants aged 18 years or older, residing in the Hail region, and willing to participate in the study. Respondents were excluded if they were under 18 years of age and/or living outside the Hail area, as well as those with incomplete submissions.

Development and application of the questionnaire

The survey was designed after conducting a comprehensive literature review and was validated by multiple urological experts. The questionnaire consisted of 15 questions divided into three main categories. The first category collected the participants' personal data. The second category focused on the frequency of barley and parsley use in the management of renal stones. The third category explored the public's attitude and perception toward the use of barley and parsley for renal stone management. To ensure language validity, the questionnaire was translated from English to Arabic by two independent translators and then back-translated from Arabic to English. Additionally, a pilot study was conducted to statistically validate the reliability of the survey using Cronbach's alpha, which returned a value greater than 0.70, indicating that the survey was reliable.

Statistical analysis

The data were extracted, revised, coded, and entered into the statistical software SPSS version 22 (Chicago, IL: SPSS Inc.). All statistical analyses were conducted using two-tailed tests. A Pearson's value of less than 0.05 was statistically significant. Descriptive analysis based on frequency and percentage distribution was done for all categorical variables including demographic data, and the frequency of barley and parsley use. Also, participants' attitudes and perceptions toward the use of barley and parsley in managing renal stones were graphed. Crosstabulation was used to assess the factors associated with the use of barley and parsley in the management of renal stones, as well as to examine the relationship between participants' use of barley and parsley and their perceptions and attitudes, using Pearson's chi-square and exact probability tests for small frequencies.

## Results

A total of 395 eligible participants completed the study questionnaire. Participants' ages ranged from 18 years to over 60 years with a mean age of 31.5±11.9 years. A total of 202 (51.1%) respondents were female, and the vast majority of them (390, 98.7%) were Saudi. Regarding educational level, 298 (75.4%) had a university level of education, 70 (17.7%) had secondary education or less, and 27 (6.8%) had a post-graduate degree. Considering profession, 308 (78%) were non-healthcare staff, while 87 (22%) were healthcare staff. Only 37 (9.4%) had a history of renal disease but 182 (46.1%) had a family history of renal stones (Table 1).

**Table 1:** Biodemographic characteristics of study participants in Hail, Saudi Arabia.

Bio-demographic data	N	%
Age (years)		
18-25	172	43.5
26-35	42	10.6
36-50	112	28.4
51-60	57	14.4
>60	12	3.0
Gender		
Male	193	48.9
Female	202	51.1
Nationality		
Saudi	390	98.7
Non-Saudi	5	1.3
Educational level		
Secondary/below	70	17.7
University level	298	75.4
Post-graduate degree	27	6.8
Profession specialty		
Non-healthcare staff	308	78.0
Healthcare staff	87	22.0
Had a renal disease history		
Yes	37	9.4
No	358	90.6
Family history of renal stones		
Yes	182	46.1
No	213	53.9

Regarding the frequency of barley and parsley used in the management of renal stones, 162 (41%) respondents used barley to treat or prevent kidney stones, which was very effective for 71 (43.8%) respondents and effective for 86 (53.1%) respondents (Table 2). Additionally, 176 (44.6%) of the respondents used parsley to treat or prevent kidney stones, which was very effective among 95 (54%) of them and effective for 71 (40.3%). Only 18 (8.2%) of barley and parsley users experienced side effects or complications. About 87 (22%) of the respondents used other home remedies to prevent kidney stones.

**Table 2:** Frequency of barley and parsley use in management of renal stones in Hail, Saudi Arabia.

Barley and parsley use	N	%
Have you used barley to treat or prevent kidney stones?		
Yes	162	41.0
No	233	59.0
If yes, how did you find the effect of barley in kidney stones? (N=162)		
Very effective	71	43.8
Somewhat effective	86	53.1
Not effective	5	3.1
Have you used parsley to treat or prevent kidney stones?		
Yes	176	44.6
No	219	55.4
If yes, how did you find the effect of parsley in kidney stones? (N=176)		
Very effective	95	54.0
Somewhat effective	71	40.3
Not effective	10	5.7
Have you experienced any side effects or complications from using barley, parsley, or other home remedies for kidney stones? (N=219)		
Yes	18	8.2
No	201	91.8
Have you ever used any other home remedies to prevent kidney stones?		
Yes	87	22.0
No	308	78.0

**Figure 1:**
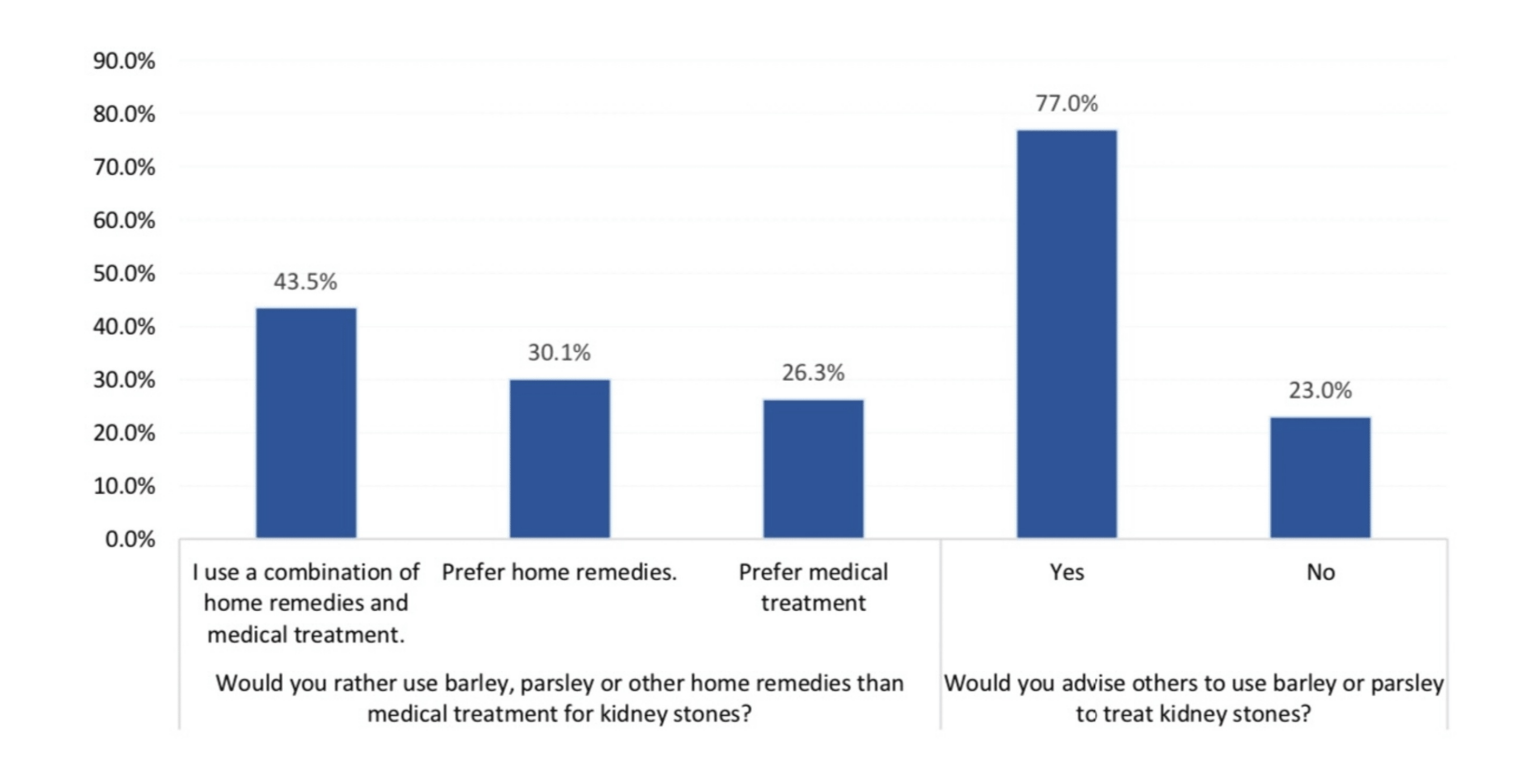
Public attitude and perception towards using barley and parsley used in the management of renal stones in Hail, Saudi Arabia.

Public attitude and perception toward the use of barley and parsley in the management of renal stones in Hail, Saudi Arabia, is presented in Figure 1. A total of 30.1% of the study respondents preferred home remedies for treating renal stones, 26.3% preferred medications, and 43.5% used a combination of both. Also, 77% reported that they would advise others to use barley or parsley to treat kidney stones.

Factors associated with using barley and parsley used in the management of renal stones in Hail, Saudi Arabia are presented in Table 3. As for barley use, it was significantly higher among older participants (85.3%) compared to 30.2% of those aged 15-25 years with a statistical significance of p=0.001. Also, 51% of females and 30.6% of males used barley for renal stones (p=0.001), 43.5% of non-healthcare staff versus 32.2% of healthcare staff used barley (p=0.048), 67.6% of those with renal disease history and 62.6% of others with a family history of renal stones used barley for renal stones (p=0.001). As for parsley, it was used for renal stones management by 66.7% of participants aged 51-60 years compared to 26.2% of others aged 18-25 years (p=0.001), and 60.4% of females compared to 28% of males (p=0.001). Likewise, 48.7% of non-healthcare workers (HCWs) used parsley versus 29.9% of HCWs (p=0.002), 67.6% of those with renal disease history used it, and 59.3% of others with a history of renal stones (p=0.001).

**Table 3:** Factors associated with the use of barley and parsley in the management of renal stones in Hail, Saudi Arabia. *P<0.05 was considered significant. **Exact probability test.

Factors	Have you used barley to treat or prevent kidney stones?		Have you used parsley to treat or prevent kidney stones?	
	N	%	N	%
Age (years)				
18-25	52	30.2	45	26.2
26-35	13	31.0	18	42.9
36-50	60	53.6	68	60.7
51-60	30	52.6	38	66.7
>60	7	58.3	7	58.3
p-Value	0.001*		0.001*	
Gender				
Male	59	30.6	54	28.0
Female	103	51.0	122	60.4
p-Value	0.001*		0.001*	
Nationality				
Saudi	158	40.5	174	44.6
Non-Saudi	4	80.0	2	40.0
p-Value	0.074**		0.837**	
Educational level				
Secondary/below	31	44.3	30	42.9
University level	122	40.9	132	44.3
Post-graduate degree	9	33.3	14	51.9
p-Value	0.616		0.715	
Profession specialty				
Non-healthcare staff	134	43.5	150	48.7
Healthcare staff	28	32.2	26	29.9
p-Value	0.048*		0.002*	
Had a renal disease history				
Yes	25	67.6	25	67.6
No	137	38.3	151	42.2
p-Value	0.001*		0.003*	
Family history of renal stones				
Yes	114	62.6	108	59.3
No	48	22.5	68	31.9
p-Value	0.001*		0.001*	

Table 4 summarizes the relation between participants' use of barley and parsley and their perception and attitude. Exactly 17.9% of barley users prefer medical treatment versus 32.2% of non-users (p=0.004). Also, 91.4% of barley users will advise others to use barley or parsley to treat kidney stones, versus 67% of non-users (p=0.001). Likewise, 33.8% of individuals who do not use parsley prefer medical treatment, compared to only 17% of those who do use parsley (p=0.001). In contrast, 93.2% of parsley users would recommend its use to others, while only 63.9% of non-users would do the same (p=0.001).

**Table 4:** Relation between participants' use of barley and parsley and their perception and attitude. *P<0.05 was considered significant.

Perception	Have you used barley to treat or prevent kidney stones?				Have you used parsley to treat or prevent kidney stones?			
	Yes		No		Yes		No	
	N	%	N	%	N	%	N	%
Would you rather use barley, parsley or other home remedies than medical treatment for kidney stones?								
I use a combination of home remedies and medical treatment	74	45.7	98	42.1	83	47.2	89	40.6
Prefer home remedies	59	36.4	60	25.8	63	35.8	56	25.6
Prefer medical treatment	29	17.9	75	32.2	30	17.0	74	33.8
p-Value	0.004*				0.001*			
Would you advise others to use barley or parsley to treat kidney stones?								
Yes	148	91.4	156	67.0	164	93.2	140	63.9
No	14	8.6	77	33.0	12	6.8	79	36.1
p-Value	0.001*				0.001*			

## Discussion

Urolithiasis is a challenging disease that affects the lifestyle of patients with high prevalence and high recurrence rates [10]. The global prevalence of urolithiasis ranged between 4% and 20% [11]; however, our study showed that the prevalence of urolithiasis among the Hail population was 9.4%. A previous study showed that the Saudi population is 2.5 times more susceptible to developing urolithiasis compared to others due to the region's climate [12]. Nonetheless, urolithiasis among the Saudi population is calcium oxalate type, which aligns with international trends [13,14]. The high prevalence and recurrence rate of urolithiasis can be reduced through metabolic and lifestyle changes [6]. In general, low sodium intake, low protein intake, and good hydration were recommended to prevent urolithiasis [15]. Non-conventional therapies are commonly used among the Saudi population to manage diseases including urolithiasis [16]. Additionally, it is not uncommon, even in developed countries. A previous study conducted in the United States of America showed that 33% of participants used at least one non-conventional therapy [17]. The review's study supports that herbal agents were significant in decreasing spares of urolithiasis but most of these studies have only involved animals or a small number of patients [18].

Regarding the use of parsley to treat or prevent urolithiasis, our data showed that 44.6% of the respondents used it. A previous study showed that parsley is a safe and effective antiurolithiasis remedy that works by decreasing calcium excretion, increasing urinary pH, and reducing protein excretion [19]. Interestingly, another study involving 20 participants investigated the effect of parsley leaf tea on preventing stone formation over two weeks and found no significant effects in urine volume, pH, uric acid, or citric acid [20].

In our study, 41% of respondents used barley to treat or prevent urolithiasis. Barley water helps encourage diuresis and maintain a regular pH of urine. In addition, high content of magnesium and vitamin B6 plays a role in preventing urolithiasis. Moreover, the antioxidants in barley prevent inflammation and infection associated with urolithiasis [16]. A study conducted by Shah et al. concluded that barley reduces and prevents urolithiasis through its diuretic effect, antioxidant, and by lowering the concentration of substances associated with urolithiasis, such as calcium, phosphate, uric acid, and oxalate [21]. Also, the protective effect is less effective than its treatment effect [22].

The use of non-conventional therapy is common around the globe, but unfortunately, there are limited studies on the efficacy of barley and parsley in managing urolithiasis. Not surprisingly, a balanced diet rich in vegetables and high-alkali fruits was recommended to protect against urolithiasis [23]. The main limitation of this study is that it is a cross-sectional study which can be subjected to a sample bias. Also, the questionnaire was not validated. Furthermore, we did not explore the frequency, formation, and quantity of barley and parsley used.

## Conclusions

In conclusion, this study highlights the prevalent use of non-conventional therapies, particularly barley and parsley. The study participants showed a high use of barley and parsley to be managing urolithiasis among the Hail population. Moreover, the most significant factors associated with using barley and parsley were older age, female gender, being a non-healthcare worker, those with a history of renal disease, and family history of renal stones. This study, therefore, finds that among the Hail population, individuals are seeking alternative approaches to manage renal stones, often prioritizing natural remedies over conventional medical treatments. This trend raises important questions about the safety and efficacy of such non-conventional therapies. Therefore, while barley and parsley may be popular among the population, it is crucial to conduct further studies to rigorously evaluate their therapeutic benefits and potential risks in the management of urolithiasis.

Ultimately, this research underscores the need for healthcare providers to acknowledge and address the use of alternative therapies in patient management, ensuring that individuals receive comprehensive care that includes evidence-based recommendations alongside their preferred treatment options.

## Appendices

**Table 5:** The electronic questionnaire used for patient data collection (15 questions).

Characteristics	Values	Responses
1. Gender	a) Female	
	b) Male	
2. Age group (years)	a) 18‑25	
	b) 26‑35	
	c) 36‑50	
	d) 51-60	
	e) >60	
3. Nationality	a) Saudi	
	b) Other	
4. Educational level	a) Primary or less	
	b) High school level	
	c) University level	
	d) Postgraduate level	
5. Occupation	a) Health worker	
	b) Non-health worker	
6. Do you have a history of renal stone disease?	a) Yes	
	b) No	
7. Do you have a family history of renal stone disease?	a) Yes	
	b) No	
8. Do you use a barley to manage or prevent renal stone?	a) Yes	
	b) No	
9. If the answer is yes, how did you find the effect of barley on kidney stones?	a) Very effective	
	b) Somewhat effective	
	c) Ineffective	
10. Do you use a parsley to manage or prevent renal stone	a) Yes	
	b) No	
11. If the answer is yes, how did you find the effect of parsley on kidney stones?	a) Very effective	
	b) Somewhat effective	
	c) Ineffective	
12. Did you experience any side effects or complications from using barley, parsley, or other home remedies for kidney stones?	a) Yes	
	b) No	
13. you ever used any other home remedies to prevent kidney stones?	a) Yes	
	b) No	
14. Do you prefer using barley, parsley, or other home remedies over medical treatment for kidney stones?	a) Yes, I prefer home remedies.	
	b) No, I prefer medical treatment.	
	c) I use a combination of home remedies and medical treatment.	
15. Would you recommend others to use barley or parsley to treat kidney stones?	a) Yes, I would recommend it.	
	b) No, I wouldn't.	

## References

[1] Sowtali SN, Ariffin SR, Nazli NS (2021). Knowledge, awareness and dietary practice on urolithiasis among general population in Kuantan, Pahang, Malaysia: preliminary findings. J Public Health Res.

[2] Amir A, Matlaga BR, Ziemba JB, Sheikh S (2018). Kidney stone composition in the Kingdom of Saudi Arabia. Clin Nephrol.

[3] Bokhari AA, Aldarwish HA, Alsanea SA (2022). Prevalence and risk factors of urolithiasis among the population of Hail, Saudi Arabia. Cureus.

[4] Safdar OY, Alzahrani WA, Kurdi MA (2021). The prevalence of renal stones among local residents in Saudi Arabia. J Family Med Prim Care.

[5] Türk C, Petřík A, Sarica K, Seitz C, Skolarikos A, Straub M, Knoll T (2016). EAU Guidelines on Interventional Treatment for Urolithiasis. Eur Urol.

[6] Clayman RV, Patel RM, Pearle M (2018). "STONE TREES": metabolic evaluation and medical treatment of the urolithiasis patient made easy. J Endourol.

[7] Bokhari AA, Aldarwish HA, Alshammari BB, Alghaslan SA, Aldhaifi SY, Alghassab AA (2022). Evaluating the level of awareness about urolithiasis among the general population of Hail, Saudi Arabia. Med Sci.

[8] Alyami FA, Rabah DM (2011). Effect of drinking parsley leaf tea on urinary composition and urinary stones′ risk factors. Saudi J Kidney Dis Transpl.

[9] Al-Yousofy F, Gumaih H, Ibrahim H, Alasbahy A (2017). Parsley! Mechanism as antiurolithiasis remedy. Am J Clin Exp Urol.

[10] Bartoletti R, Cai T, Mondaini N, Melone F, Travaglini F, Carini M, Rizzo M (2007). Epidemiology and risk factors in urolithiasis. Urol Int.

[11] Liu Y, Chen Y, Liao B, Luo D, Wang K, Li H, Zeng G (2018). Epidemiology of urolithiasis in Asia. Asian J Urol.

[12] Khan AS, Rai ME, Gandapur Gandapur, Pervaiz A, Shah AH, Hussain AA, Siddiq M (2004). Epidemiological risk factors and composition of urinary stones in Riyadh Saudi Arabia. J Ayub Med Coll Abbottabad.

[13] Mosli HA, Mosli HH, Kamal WK (2013). Kidney stone composition in overweight and obese patients: a preliminary report. Res Rep Urol.

[14] (2020). Prevalence and characterization of urolithiasis in the western region of Saudi Arabia. Urol Ann.

[15] Porena M, Guiggi P, Micheli C (2007). Prevention of stone disease. Urol Int.

[16] Kamal WK, Bokhari A, Alesia SM, Mahjari TM, Binsalman WA, Laher AE, Adam A (2024). Utilization of barley and parsley for the management of urolithiasis among the Saudi Arabian population. Urol Ann.

[17] Eisenberg DM, Kessler RC, Foster C, Norlock FE, Calkins DR, Delbanco TL (1993). Unconventional medicine in the United States. Prevalence, costs, and patterns of use. N Engl J Med.

[18] Emiliani E, Jara A, Kanashiro AK (2021). Phytotherapy and herbal medicines for kidney stones. Curr Drug Targets.

[19] Saeidi J, Bozorgi H, Zendehdel A, Mehrzad J (2012). Therapeutic effects of aqueous extracts of Petroselinum sativum on ethylene glycol-induced kidney calculi in rats. Urol J.

[20] Alyami FA, Rabah DM (2011). Effect of drinking parsley leaf tea on urinary composition and urinary stones' risk factors. Saudi J Kidney Dis Transpl.

[21] Shah JG, Patel BG, Patel SB, Patel RK (2012). Antiurolithiatic and antioxidant activity of Hordeum vulgare seeds on ethylene glycol-induced urolithiasis in rats. Indian J Pharmacol.

[22] Rashid S, Sameti M, Alqarni MH, Abdel Bar FM (2023). In vivo investigation of the inhibitory effect of Peganum harmala L. and its major alkaloids on ethylene glycol-induced urolithiasis in rats. J Ethnopharmacol.

[23] Prezioso D, Strazzullo P, Lotti T (2015). Dietary treatment of urinary risk factors for renal stone formation. A review of CLU Working Group. Arch Ital Urol Androl.

